# Supplementary material for: A protocol to establish and maintain organotypic cerebellar slice culture (OCerSC) from aged mice
Source: PLoS One. 2026 Apr 17;21(4):e0342373. doi: 10.1371/journal.pone.0342373 (PMC13089748; doi:10.1371/journal.pone.0342373)
Supplement: S1 File — (PDF) [file pone.0342373.s001.pdf]

Aug 13, 2024

Establishment and Maintenance of Organotypic Cerebellar Slice Cultures (OCerSC) from Aged Mice

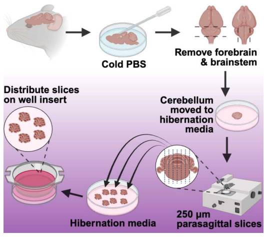

DOI
dx.doi.org/10.17504/protocols.io.q26g71428gwz/v1

Kaitlan S. Smith[1], Michael Fernandes de Almeida[1], Jonathan C Schisler[1]

[1]The University of North Carolina at Chapel Hill

Kaitlan S. Smith: Department of Pharmacology and McAllister Heart Institute
Michael Fernandes de Almeida: Department of Pharmacology and McAllister Heart Institute
Jonathan C Schisler: Department of Pharmacology, Department of Pathology and Lab Medicine, and McAllister Heart Institute

Michael Fernandes de Almeida
University of North Carolina

OPEN ACCESS

DOI: dx.doi.org/10.17504/protocols.io.q26g71428gwz/v1

Protocol Citation: Kaitlan S. Smith, Michael Fernandes de Almeida, Jonathan C Schisler 2024. Establishment and Maintenance of Organotypic Cerebellar Slice Cultures (OCerSC) from Aged Mice. **protocols.io** https://dx.doi.org/10.17504/protocols.io.q26g71428gwz/v1

License: This is an open access protocol distributed under the terms of the **Creative Commons Attribution License**, which permits unrestricted use, distribution, and reproduction in any medium, provided the original author and source are credited

Protocol status: Working
We use this protocol and it's working

Created: May 10, 2024

Last Modified: August 13, 2024

Protocol Integer ID: 99579

Keywords: organotypic brain slice culture, aging, cerebullum, adult mice, in vivo

Funders Acknowledgement:
Jonathan Schisler
Grant ID: R01AG066710
Jonathan Schisler
Grant ID: R01AG061188

Disclaimer

NA.

Abstract

This protocol outlines a method for culturing organotypic slices from aged mouse cerebellar tissue, maintaining the cerebellum's complex three-dimensional structure and cellular composition ex vivo. This technique simulates various physiological and pathophysiological conditions to study neurodevelopmental and neurodegenerative disorders and synaptic plasticity mechanisms. Expected outcomes include detailed neuronal morphology and functional connectivity analyses at the cellular and circuit levels using advanced imaging, electrophysiological techniques, and biochemical analyses such as protein expression, gene expression, metabolic activity, oxidative stress markers, neurotransmitter levels, calcium imaging, and enzyme activity assays.

Image Attribution

Created with BioRender.com. Agreement number QT275WKSOG and AH275WKSRI.

Guidelines

When planning for this experiment assume that one six-well plate, with 4-6 slices per well, can generally accommodate cerebellum slices for 2 animals.

Materials

Elmers Rubber Cement
Glutamax (100x)Gibco - Thermo FischerCatalog #35050-061
Neurobasal-A MediumCatalog #10888022
Corning™ Penicillin-Streptomycin SolutionFisher ScientificCatalog #MT30002CI
Nunc™ Cell-Culture Treated Multidishes, 6 well Thermo FisherCatalog #140685
Glucose SolutionThermo FisherCatalog #A2494001
Whatman Grade 1 Qualitative Filter PapersCytivaCatalog #1001-055
Indomethacin 1 g STEMCELL Technologies Inc.Catalog #73942
Blades for McIlwain Tissue Chopper Campden InstrumentsCatalog #Model TC752-1
Millicell Cell Culture Insert, 30 mm, hydrophilic PTFE, 0.4 µm, Hydrophilic PTFEMerck MilliporeSigma (Sigma-Aldrich)Catalog #PICM03050
Amphotericin B solution-Merck MilliporeSigma (Sigma-Aldrich)Catalog #A2942
Petri Dishes with Clear Lid, Raised ridgeThermo FisherCatalog #FB0875712
Hibernate™-A MediumThermo ScientificCatalog #A1247501

Protocol materials

- Indomethacin 1 g **STEMCELL Technologies Inc. Catalog #**73942 Step 3
- Petri Dishes with Clear Lid, Raised ridge **Thermo Fisher Catalog #**FB0875712 Step 1
- Glucose Solution **Thermo Fisher Catalog #**A2494001 Step 3
- Neurobasal-A Medium **Catalog #**10888022 Step 3
- Hibernate™-A Medium **Thermo Scientific Catalog #**A1247501 Step 3
- B-27™ Supplement (50X), serum free **Gibco - Thermo Fisher Catalog #**17504044 Step 3
- Millicell Cell Culture Insert, 30 mm, hydrophilic PTFE, 0.4 µm, Hydrophilic PTFE **Merck MilliporeSigma (Sigma-Aldrich) Catalog #**PICM03050 Step 1
- Corning™ Penicillin-Streptomycin Solution **Fisher Scientific Catalog #**MT30002CI Step 3
- N2 supplement (100x supplement) **Gibco, ThermoFisher Catalog #**17502048 Step 3
- BupH Phosphate Buffered Saline Packs **Thermo Fisher Scientific Catalog #**PI28372 Step 3
- Glutamax (100x) **Gibco - Thermo Fischer Catalog #**35050-061 Step 3
- Elmers Rubber Cement Step 1
- Blades for McIlwain Tissue Chopper **Campden Instruments Catalog #**Model TC752-1 Step 1
- Amphotericin B solution **Merck MilliporeSigma (Sigma-Aldrich) Catalog #**A2942 Step 3
- Whatman Grade 1 Qualitative Filter Papers **Cytiva Catalog #**1001-055 Step 1
- Nunc™ Cell-Culture Treated Multidishes, 6 well **Thermo Fisher Catalog #**140685 Step 1

Safety warnings

It is not recommended that animal dissection and cerebellum slicing be done under the same hood. Animals should be dissected on a laboratory bench or in a separate hood.

Risk of Contamination:

- Performing dissection and slicing under the same hood increases the risk of cross-contamination, which can compromise the sterility and quality of the cerebellum slices.

Ethics statement

This protocol adheres to the ethical guidelines and regulations set forth by the Institutional Animal Care and Use Committee at the University of North Carolina at Chapel Hill. All animal procedures follow the principles of humane treatment and care.

The use of isoflurane for euthanasia was chosen to minimize stress and discomfort for the animals, ensuring a humane and ethical approach. All personnel involved in the experiment are trained in proper handling and care techniques to ensure the welfare of the animals.

This protocol (Protocol Number: 22-256) was reviewed and approved by the relevant ethical committee, and all necessary permits and approvals have been obtained. Continuous monitoring and assessment are conducted to ensure compliance with ethical standards throughout the experiment.

Before start

Before you begin the slicing process, ensure that all six-well plates are prepped and placed in the incubator at 37 °C with 5% CO_2 for at least 30 minutes. This step is crucial to ensure that the media and inserts are at the correct temperature and conditions, which helps maintain the viability and quality of the cerebellum slices.

Safety Precautions:

- When handling the plates, incubator, animals, and equipment, wear appropriate personal protective equipment (PPE), such as gloves, safety eyewear, and a lab coat.
- Ensure that dissection is performed on a laboratory bench or in a separate hood dedicated to dissection.
- Use a different hood or area for cerebellum slicing to maintain a sterile environment.
- Follow proper aseptic techniques to minimize the risk of contamination and maintain sterility.

Materials

1 **Slice Culture Consumables:**

- Millicell Cell Culture Insert, 30 mm, hydrophilic PTFE, 0.4 µm, Hydrophilic PTFE **Merck MilliporeSigma (Sigma-Aldrich) Catalog #**PICM03050
- Whatman Grade 1 Qualitative Filter Papers **Cytiva Catalog #**1001-055
- Nunc™ Cell-Culture Treated Multidishes, 6 well **Thermo Fisher Catalog #**140685
- Elmers Rubber Cement **Contributed by users**
- Blades for McIlwain Tissue Chopper **Campden Instruments Catalog #**Model TC752-1
- Petri Dishes with Clear Lid, Raised ridge **Thermo Fisher Catalog #**FB0875712

1.1 **Tools:**

- Scalpel
- Forceps
- Razor blade
- Scissors
- Transfer pipettes
- Paint brushes (recommended sizes no.3 and no.2)

Equipment

2

Equipment	
**Biological safety cabinet**	NAME
AC2-4S8-TU	TYPE
Esco	BRAND
303110	SKU
https://analis.be/fr-be/sample-handling-in-ivf-labs/ac2-4s8-tu-airstream-plus-class-ii-biological-303110	LINK

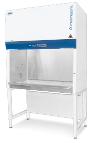

Equipment	
**Forma™ Series II Water-Jacketed CO2 Incubator, 184L**	NAME
CO2 Incubators	TYPE
Thermo Scientific™	BRAND
3110	SKU
https://www.thermofisher.com/order/catalog/product/3110	LINK

Equipment	
McIlwain Tissue Chopper with Petri Dish Modification	NAME
Tissue chopper	TYPE
Campden Instruments	BRAND
Model TC752-PD	SKU

Reagents

3
- Amphotericin B solution **Merck MilliporeSigma (Sigma-Aldrich) Catalog #**A2942
- Corning™ Penicillin-Streptomycin Solution **Fisher Scientific Catalog #**MT30002CI
- Glutamax (100x) **Gibco - Thermo Fischer Catalog #**35050-061
- Glucose Solution **Thermo Fisher Catalog #**A2494001
- Indomethacin 1 g **STEMCELL Technologies Inc. Catalog #**73942
- Neurobasal-A Medium **Contributed by users Catalog #**10888022
- Hibernate™-A Medium **Thermo Scientific Catalog #**A1247501
- B-27™ Supplement (50X), serum free **Gibco - Thermo Fisher Catalog #**17504044
- N2 supplement (100x supplement) **Gibco, ThermoFisher Catalog #**17502048
- BupH Phosphate Buffered Saline Packs **Thermo Fisher Scientific Catalog #**PI28372

Solutions

4 **Slice Culture Media #1**

Neurobasal-A Medium supplemented with:
- 2% B27
- 1% N2 Supplement
- 1% Glutamax
- 200 g/L glucose
- 1.5% Penicillin/streptomycin
- 1.5% Amphotericin B

- 80 µM Indomethacin

Slice Culture Media #2

Neurobasal-A Medium supplemented with:

- 2% B27
- 1% N2 Supplement
- 1% Glutamax
- 200 g/L glucose
- 1.5% Penicillin/streptomycin
- 1.5% Amphotericin B

Phosphate Buffered Saline

Preparation Steps

5 **Before Starting Tissue Harvest and Slicing Steps**

Only one cerebellum should be simultaneously sliced and harvested to minimize the time between cerebellum isolation and slice distribution to the inserted wells. This practice helps ensure the viability and quality of the cerebellum slices by reducing the time the tissue is exposed to non-optimal conditions.

Safety information

- When handling ethanol, plates, media, and animals, wear appropriate personal protective equipment (PPE), such as gloves, a lab coat, and safety goggles.
- Ensure proper ventilation in the workspace to avoid inhaling fumes.
- Ensure proper aseptic techniques to maintain sterility.

5.1 **Sterilize Equipment:**

- Use 70% ethanol to wipe down all surfaces inside the biological safety cabinet thoroughly. Ensure all areas, including corners and hard-to-reach spots, are properly cleaned.
- Apply 70% ethanol to a clean cloth or paper towel and wipe down the tissue slicer and all dissection instruments, ensuring that all surfaces are clean.
- Allow the equipment to air dry completely before use.

5.2 **Prepare Six-well Plates** 30m

- Place one cell culture insert per well and add 1mL of Slice Culture Media #1.
- Ensure membranes are thoroughly wet and that there are no air bubbles.
- Place the six-well plates in an incubator at 37 °C with 5% CO_2 for at least 30 minutes before starting the cerebellar slicing process.

5.3 **Prepare Tissue Slicer**

- Mount a new blade on the tissue slicer.

- Set the slice thickness 250 µm .

5.4 **Prepare the Chopping Disc**

- Using Elmer's glue, attach Whatman filter paper onto the chopping disc.
- Allow the glue to dry completely to ensure the filter paper is firmly attached.
- Mount the chopping disc onto the tissue slicer.

5.5 **Prepare Hibernation Media Dishes**

- Place Hibernate-A Medium in a 37 °C water bath for at least 30 minutes before starting the cerebellar slicing process. Ensure the medium reaches the correct temperature to maintain tissue viability.
- Prepare one 60 mm petri dish per animal.
- Add 4-5 mL of pre-warmed Hibernate-A Medium to each dish.

5.6 **Prepare Euthanasia and Dissection Prep**

- Set up the euthanasia chamber according to the approved animal protocol.
- Ensure that all necessary components are in place and functioning correctly.
- Double-check that the sterile dissection tools are ready.
- Obtain a 66 mm sterile petri dish for use during the dissection process.
- Prepare sterile PBS and set to chill on ice.
- Transport the mice to the dissection area. This protocol was developed using 22-92 week-old B6129F1/Tac mice.

> **Note**
>
> This protocol uses isoflurane to euthanize the animals. While other euthanasia methods, such as CO_2, are feasible, isoflurane is recommended because it is less stressful for the animals and allows for the quick removal of the brain, ensuring that the tissue remains viable.

Cerebellar Slicing

6

> **Note**
>
> **Recommendation:** To ensure the viability and quality of the cerebellum slices, slice only one cerebellum at a time. This minimizes the time between cerebellum isolation and the distribution of slices into the wells, thereby preserving their structural integrity and functionality.
>
> ***Optional:*** A dissection microscope is recommended to guarantee that only intact cerebellum slices are selected. Using a microscope can help identify and exclude any damaged or incomplete slices, thereby improving the overall quality of the samples.

6.1 **Dissect the Brain:**

1. Following proper IACUC-approved animal euthanasia, decapitate the animal.
2. Carefully expose and remove the brain, taking special care not to damage the cerebellum.
3. Transfer the brain to an empty sterile petri dish.
4. Using a transfer pipette, wash the brain with cold, sterile PBS to remove any extraneous hair or blood (**Figure 1**).
5. Remove the forebrain and spinal cord to isolate the cerebellum (**Figure 1**).
6. Transfer the cerebellum to a sterile petri dish containing 4-5 ml of pre-warmed Hibernate media (**Figure 1**).

6.2 **Slicing the Cerebellum:**

1. Transfer the cerebellum to the Whatman filter paper glued onto the chopping disc.
2. Critical Point: Orient the brain so the cerebellum faces upwards and the sagittal plane is aligned with the chopper blade (**Figure 1**). This ensures that the slices will be sagittal, maintaining the structural integrity necessary for subsequent analyses.
3. Using the manual setting on the chopper, slice the cerebellum into 250 µm sections (**Figure 1**).
4. Use a paintbrush to hydrate the cerebellar tissue throughout the slicing process. (Avoid overwetting the cerebellum and the filter paper).
5. After each cut, use a paintbrush to transfer the cerebellum slice to a petri dish filled with pre-warmed Hibernate media (**Figure 1**).
6. Select the intact cerebellar slices and transfer 4 to 6 individual slices onto the insert membranes in the six-well plates (**Figure 1**)to distribute them evenly on the insert.
7. Incubate at 37 °C with 5% CO_2.

Note

When distributing the cerebellar slices onto the membranes, ensure that the slices are not close to the insert wall or touching one another. We recommend 4-6 slices per well for adult mice. Avoid the accumulation of media on the top of the membrane insert; if needed, remove excess medium carefully with a transfer pipette to secure the tissue's attachment to the insert membrane. A wet paintbrush can also be utilized to place the slices in an appropriate configuration following the removal of excess media.

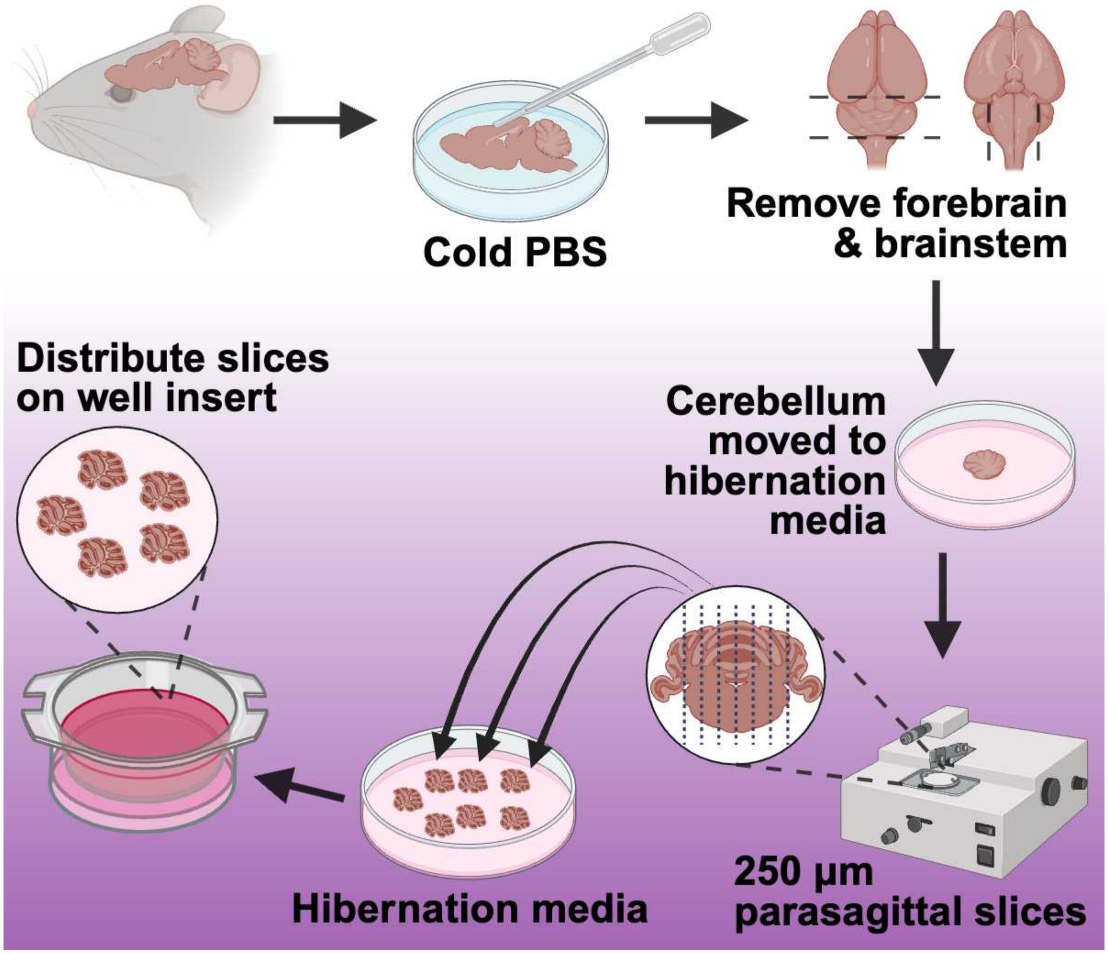

Figure 1. Workflow for Preparing Organotypic Cerebellar Slice Cultures. This schematic illustrates the process of preparing organotypic cerebellar slice cultures. The procedure begins with extracting the brain from an aged mouse, followed by a rinse with cold PBS. The cerebellum is then separated from the forebrain and brainstem and placed in hibernation media. When ready for sectioning, the cerebellum is oriented in a parasagittal manner and sliced into 250 µm thick sections. These slices are returned to the hibernation media until they are ready to be positioned on the well insert. Care should be taken to distribute 4 to 6 cerebellar slices on the insert evenly, avoiding placement too close to the insert wall. This method ensures the preservation of tissue integrity and functionality for subsequent analyses. Figure created using Biorender.com.

Culture Conditions

5d

7

5d

Note

Our protocol proposes a novel approach 18-24 hours after plating, with subsequent options to extend the ex vivo lifespan of slices from adult cerebellar tissue to at least 14 days (**Figure 2**). This is achieved by significantly altering the medium composition during the culture period. These alterations increase tissue viability and promote the maintenance of well-preserved cerebellar slices, enabling improved biomolecular analysis such as protein expression levels, immunofluorescence, and measurement of released enzymes.

Incubation and Media Changes

1. *Initial Incubation:* Once slices are securely placed onto the membrane, incubate plates at 37ºC with 5% CO_2. 37 °C
2. *First Media Change (18-24 hours after slicing):* Remove the slice culture media #1 and replace it with fresh slice culture media #1. 24:00:00
3. *Subsequent Media Changes:* After the first media change, change the culture media every 2-3 days.
4. *Media Schedule:*

- **DEV 1-5:** Maintain the OCerSC in Slice Culture Media #1, following a scheduled media change every 2-3 days. 48:00:00
- **DEV6 - End of the experiment:** Maintain the OCerSC in Slice Culture Media #2, with a scheduled media change every 2-3 days. 48:00:00

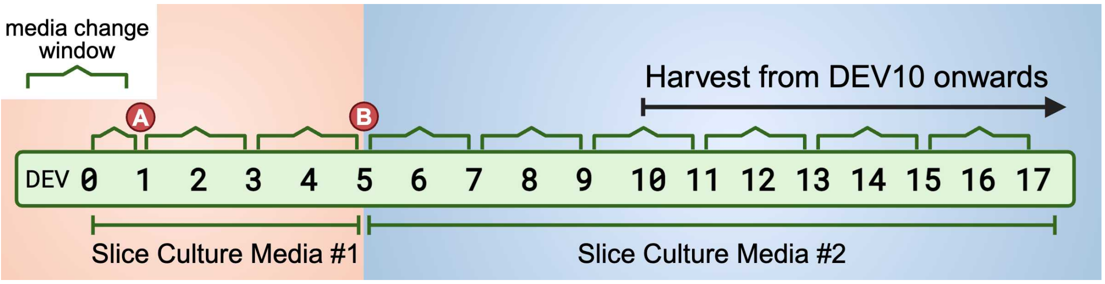

Figure 2: Timeline of Organotypic Cerebellar Slice Culture Maintenance. This figure presents a detailed timeline for maintaining organotypic cerebellar slice cultures (OCerSc) throughout up to 16 days ex vivo (DEV). Initially, OCerSc is sustained in Slice Culture Media #1 for five days post-plating, followed by a transition to Slice Culture Media #2 from days six through sixteen. The media change window highlights the critical period for media changes. The circled points A and B represent the importance of the first media change 18-24 hours after plating and the change to Slice Culture Media #2, respectively. Subsequent options after the timeline include harvesting slices or continuing maintenance every 48 hours. Figure created using Biorender.com.

Expected result

Objective: Maintain the structural integrity, contamination-free status, and viability of organotypic cerebellar tissue during in vitro culture.
Indicators of Success:

- **Clear Structure:** The cerebellar slices should exhibit a well-defined and intact structure.
- **No Damage:** There should be no visible signs of physical damage or tearing in the slices.
- **No Contamination:** The slices should be free from any signs of contamination, such as discoloration or unexpected growth.
- **Flattening Process:** During in vitro development, the tissue is expected to flatten due to sagittal cuts in the cerebellum slices, indicating the activation of an inflammatory pathway. This process helps in the recovery and elimination of dead cells.
- **Tissue Swelling:** Swelling of the tissue, which leads to flattening, should be observed, particularly during the first 10 days in vitro.
- **Enhanced Optical Properties:** Flattened slices should exhibit better optical properties than brain slices prepared acutely.

Quality Checks:

- **Microscopic Examination:** Use a microscope to inspect the slices for structural integrity and absence of damage.
- **Sterility Confirmation:** Ensure that the slices remain free from contamination by regularly checking for any signs of microbial growth.
- **Regular Monitoring:** Consistently observe the tissue for signs of flattening and swelling to ensure proper development in vitro.
- **Optical Examination:** Verify that the slices maintain enhanced optical properties, indicating successful preparation and maintenance.
- **Optional Tests:** Collect culture media from each well periodically for cell viability assays (e.g., MTT assay) or cytotoxicity tests (e.g., LDH). Additionally, control wells can be submitted to Propidium Iodide staining over time to assess cell death levels.

Protocol references

Kim H, Kim E, Park M, Lee E, Namkoong K. Organotypic hippocampal slice culture from the adult mouse brain: a versatile tool for translational neuropsychopharmacology. Prog Neuropsychopharmacol Biol Psychiatry. 2013;41:36-43. doi:10.1016/j.pnpbp.2012.11.004

Stoppini L, Buchs PA, Muller D. A simple method for organotypic cultures of nervous tissue. J Neurosci Methods. 1991;37(2):173-182. doi:10.1016/0165-0270(91)90128-m
