# Supplementary material for: A protocol to establish and maintain organotypic cerebellar slice culture (OCerSC) from aged mice
Source: PLoS One. 2026 Apr 17;21(4):e0342373. doi: 10.1371/journal.pone.0342373 (PMC13089748; doi:10.1371/journal.pone.0342373)
Supplement: S2 File — (PDF) [file pone.0342373.s002.pdf]

SUPPORTING INFORMATION FILE 2

A protocol to establish and maintain organotypic cerebellar slice culture (OCerSC) from aged mice

Michael F. Almeida[1¶], Kaitlan Smith[1,2¶], Michael A. Garris[1], Rebekah Sanchez-Hodge[1], Meagan Colie[1,2], and Jonathan C. Schisler[1-3*]

[1] The McAllister Heart Institute, The University of North Carolina at Chapel Hill, Chapel Hill, North Carolina, United States of America

[2] Department of Pharmacology, The University of North Carolina at Chapel Hill, Chapel Hill, North Carolina, United States of America

[3] Department of Pathology and Lab Medicine, and Computational Medicine Program, The University of North Carolina at Chapel Hill, Chapel Hill, North Carolina, United States of America

¶ These authors contributed equally to this work

* Corresponding author

Email: schisler@unc.edu (JCS)

Funding: This work was supported by the National Institute of Aging R01AG066710 and R01AG061188 to JCS and P30AG072958 to MFA. The content is solely the authors' responsibility and does not necessarily represent the official views of the National Institutes of Health.

Competing interests: The authors have declared that no competing interest exists.

Data availability: Not applicable.

Associated content: dx.doi.org/10.17504/protocols.io.q26g71428gwz/v1

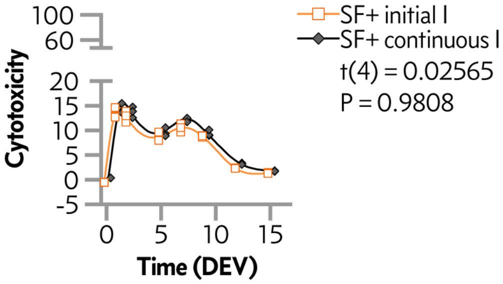

Supplementary Figure 1. Prolonged indomethacin exposure does not further reduce cytotoxicity beyond the initial 5 days in culture. Cytotoxicity was monitored ex vivo (DEV) for 15 days by measuring lactate dehydrogenase (LDH) release into the culture medium using the LDH-Glo™ Cytotoxicity Assay (Promega, J2380) according to the manufacturer's instructions. Media were collected at each complete media change and stored in the LDH storage buffer until analysis. Data are presented as a spline plot with overlying dot plots showing individual measurements at each time point (N = 3 biological replicates per day). Symbols represent treatment groups: ◇ SFM supplemented with 80 μM indomethacin continuously for 15 DIV (SF+ continuous I) □ SFM supplemented with 80 μM indomethacin for the first 5 DIV only, followed by SFM alone until 15 DIV (SF+ initial I). To compare overall cytotoxicity across the full culture period, the area under the curve (AUC) was calculated for each biological replicate. Mean AUC ± SD: SF+ initial I: 120.8 ± 6.8 (n = 3) SF+ continuous I: 120.7 ± 6.9 (n = 3). No significant difference was observed between groups (unpaired two-tailed t-test: $t(4) = 0.02565$, $P = 0.9808$, ns). Variances were also comparable (F test: $F(2,2) = 1.044$, $P = 0.9786$).

Group	AUC (mean)	Standard Deviation
SF	365.067	72.813
SF+I	213.100	30.259
HS	872.700	14.962
HS+I	762.300	116.516

Supplementary Table 1. Mean and standard deviation of the area under the curve (AUC) values for cytotoxicity from Figure 1, based on LDH measurements.
