# Supplementary material for: A protocol to establish and maintain organotypic cerebellar slice culture (OCerSC) from aged mice
Source: PLoS One. 2026 Apr 17;21(4):e0342373. doi: 10.1371/journal.pone.0342373 (PMC13089748; doi:10.1371/journal.pone.0342373)
Supplement: Attachment — Submitted filename: Almedia Slice Response.docx [file pone.0342373.s004.docx]

# Point-by-Point Response to Reviewers

Dear Editors and Reviewers,

Thank you for your insightful and constructive feedback on our manuscript, "A protocol to establish and maintain organotypic cerebellar slice culture (OCerSC) from aged mice" (Manuscript ID: PONE-D-24-43970). We have thoroughly considered all comments and conducted additional experiments to address the concerns raised. The manuscript has been significantly revised to include new data, higher-resolution images, and additional references, while adhering to *PLOS ONE*’s Lab Protocol Article Template. Below, we provide a point-by-point response to the editor’s requirements and reviewers’ comments, highlighting the changes made to strengthen the manuscript. We believe these revisions enhance the clarity, rigor, and scientific value of our protocol.

## Response to Editor’s Requirements

1. Use of PLOS ONE template for protocols:

We have reformatted the manuscript to strictly adhere to the ***PLOS ONE* Lab Protocol Article Template**, ensuring that all required sections (Title, Abstract, Introduction, Materials and Methods, Expected Results, Ethics Declarations, Supporting Information, Acknowledgments, Authors’ Contributions, References) are included. The step-by-step protocol is provided as Supporting Information file S1 and is hosted on protocols.io (dx.doi.org/10.17504/protocols.io.q26g71428gwz/v1).

2. Details on animal experiments:

We apologize for the oversight in our initial submission. The Materials and Methods section has been updated to include detailed information on animal experiments (page 4). Specifically, we have clarified: (1) **Methods of anesthesia and euthanasia**: Mice were anesthetized and euthanized using isoflurane (1–3% in oxygen, drop method, as needed) via inhalation, followed by cervical dislocation to ensure death. Death was confirmed by testing footpad reflexes, in accordance with the recommendations of the Panel on Euthanasia of the American Veterinary Medical Association. (2) **Efforts to alleviate suffering**: Hydrogel and moistened chow were provided for mice with reduced mobility to ensure access to food and water. Animals exhibiting critical health deterioration (e.g., head tucking, skin lesions, inability to walk/drink/eat) were promptly euthanized per IACUC guidelines to minimize suffering. These additions reflect our commitment to ethical conduct and animal welfare, and we thank the editor for emphasizing their importance.

3. Inclusion of "Protocol" in the title:

The manuscript title has been revised to "A protocol to establish and maintain organotypic cerebellar slice culture (OCerSC) from aged mice," which clearly reflects its nature as a protocol and aligns with the editor’s recommendation.

4. Data sharing requirements:

Thank you for bringing this issue to our attention. The phrase "data not shown" in our initial submission was an oversight and has been removed from the manuscript to comply with PLOS ONE’s data-sharing policy. We apologize for any confusion this may have caused. The manuscript now focuses on the 14-day viability of organotypic cerebellar slice cultures (OCerSC) from aged mice, with all relevant data fully presented in the Expected Results section (pages 5-6), supported by lactate dehydrogenase (LDH) release assays, western blot analysis of calbindin expression, and immunofluorescence imaging. We appreciate the editor’s attention to this detail, which has enhanced the clarity and transparency of our submission.

## Response to Reviewer #1

1. Manuscript structure and content:

**Reviewer Comment**: “The structure of the paper is confusing. It would improve the publication if the authors included a proper results and discussion section. Challenges and hypotheses should be included in the discussion and the results need to be critiqued against the literature.”

We appreciate the reviewer’s feedback on the manuscript’s structure. As a Lab Protocol, our manuscript follows *PLOS ONE*’s specific *Lab Protocol Article Template*, which includes an “Expected Results” section instead of traditional “Results” and “Discussion” sections. We contacted the editor for clarification, and they confirmed that this format is appropriate (see correspondence with Ethan Krajewski provided below). To address the reviewer’s concerns, we have reorganized the manuscript for clarity and expanded the Introduction (pages 2-3) to include challenges (e.g., the truncated lifespan of adult cerebellar slices ex vivo, median 7 days) and hypotheses (e.g., that media modifications can enhance viability). The Expected Results section (pages 5-6) now critiques our findings against the literature, comparing our 14-day viability with prior studies (e.g., [9, 16-24]). We believe these changes align with the reviewer’s suggestions while adhering to the journal’s guidelines.

2. Bibliographic information:

**Reviewer Comment**: “In general, lots of bibliographic information for the sources of the information provided in the manuscript is missing.”

We thank the reviewer for highlighting this. We have thoroughly revised the manuscript to ensure all statements are supported by appropriate references. New citations have been added throughout the Introduction (pages 2-3) and Expected Results (pages 5-6) to contextualize our protocol within the literature, including references on organotypic slice cultures (e.g., [8, 52, 53]) and the anti-inflammatory effects of indomethacin (e.g., [31, 35, 36]). These additions enhance the scientific rigor and clarity of the manuscript.

3. Figure 1:

**Reviewer Comment**: “The authors should highlight in figure 1 the dot for their method.”

We appreciate the reviewer for this suggestion. To address it and prevent potential misinterpretation of data from other studies, we have updated Figure 1 to include a data point from our current study, represented by the ◪ symbol (labeled Cb‡), indicating a 14-day viability for our protocol. Horizontal lines indicate median DEV values (22 days for neonatal, 7 days for adult), and ordinal logistic regression confirms a reduction in the odds of longer survival with increasing age.

4. Representative images and cerebellar nuclei:

**Reviewer Comment**: “The representative images should be exchanged, higher magnification need to be used, in order to better see the morphology. Are the cerebellar nuclei in the slices? In all of them?”

We thank the reviewer for this valuable feedback. We have replaced all representative images with higher-resolution versions, including brightfield images in Figure 2 and higher-magnification (20x and 40x) confocal images in Figure 3 to clearly illustrate single-cell morphology. In Figure 3, the high-resolution tiled overview image in 3A showcases the cerebellar structure, while panels 3C and 3E provide detailed views of the three layers—the molecular layer (ML), Purkinje cell layer (PC), and granule cell layer (GL)—highlighting their distinct morphology. Our parasagittal slices (250–400 µm thick) can capture the deep cerebellar nuclei (DCN, dentate, interposed, fastigial) if desired, particularly in the medial and intermediate sections, as confirmed by DAPI staining, which reveals large neurons in the deep white matter. However, not all slices contain DCN due to mediolateral positioning; lateral slices primarily capture the cerebellar cortex. Staining includes calbindin to highlight Purkinje cell axons projecting to the DCN and DAPI to visualize DCN neurons, as detailed in the updated Materials and Methods (pages 3-5) and Figure 3 legend. The improved resolution and layering details in Figure 3 address the reviewer’s request for better visualization of morphology. Additionally, we have included western blot analysis of calbindin expression (Figure 2C), which validates Purkinje cell integrity across conditions, further supporting our morphological findings.

5. Neuronal circuit functionality:

**Reviewer Comment**: “Authors need to demonstrate the functionality of neuronal circuits to demonstrate (partial) neuronal functionality of the slices.”

We appreciate this suggestion. To demonstrate partial neuronal functionality, we have strengthened our evidence with calbindin immunostaining (Figure 3) and Western blot analysis (Figure 2C). These techniques show preserved Purkinje cell morphology and robust calbindin expression in serum-free plus indomethacin (SF+I) conditions at 14 days ex vivo (DEV), indicating maintained cellular health. Western blot analyses quantify calbindin levels across conditions, with SF+I showing higher expression. Additionally, Figure 3E details the intricate dendritic arborization of Purkinje cells, while Figure 3D reveals intact Purkinje cell layers, further supporting structural preservation critical to circuit functionality. Given Purkinje cells’ pivotal role in cerebellar circuits, this structural integrity suggests partial neuronal functionality. Moreover, LDH release assays (Figure 2B) confirm reduced cytotoxicity in SF+I conditions, supporting overall slice viability. Together, these data provide robust evidence of partial neuronal functionality, establishing a strong foundation for this protocol. This platform also offers opportunities for future studies to explore functional connectivity using advanced electrophysiological techniques, enhancing its utility in neurobiological research.

## Response to Reviewer #2

1. Protocol effectiveness and Figure 3B:

**Reviewer Comment**: “Figure 3B… does not satisfactorily show the dendrite arborization of Purkinje cells… there are no data supporting author’s conclusion (that is indomethacin has a beneficial effect on the survival and morphology of Purkinje cells).”

We thank the reviewer for their insightful feedback. We have replaced Figure 3B with higher-resolution (40x) confocal images in Figure 3, which display well-preserved Purkinje cell dendritic arborization in serum-free plus indomethacin (SF+I) conditions at 14–16 days ex vivo (DEV). This enhancement is enabled by the improved optical clarity observed in Figure 2D (brightfield images), which provides a solid foundation for generating these high-resolution images. Notably, in the absence of indomethacin, the tissue remains swollen, rendering imaging methods ineffective and obscuring detailed morphology, emphasizing indomethacin’s critical role. To support our conclusion that indomethacin benefits not only Purkinje cells but also the overall cerebellar tissue, we present quantitative evidence from lactate dehydrogenase (LDH) release assays (Figure 2B), showing a 71% reduction in cytotoxicity in SF+I conditions compared to horse serum (HS) conditions, indicating improved viability across cell types. Additionally, the new western blot analysis of calbindin expression (Figure 2C) demonstrates higher calbindin levels in SF+I conditions, validating the health of Purkinje cells. Meanwhile, the preserved tissue structure in Figure 3 benefits granule cells and other layers. These revisions provide compelling visual and quantitative evidence of our protocol’s broad effectiveness.

2. Quantitative data and additional quantification methods:

**Reviewer Comment**: “The authors should present a graph (with appropriate statistical tests) comparing the survival of Purkinje cells in the presence or absence of indomethacin…”

We sincerely thank the reviewer for their detailed feedback and valuable suggestions for enhancing our manuscript. We acknowledge the request for a graph with statistical tests comparing Purkinje cell survival in the presence or absence of indomethacin, along with the suggestions for additional quantification methods and improved image quality. Below, we address each point, utilizing existing data to demonstrate the protocol’s effectiveness while aligning with the scope of a *PLOS ONE* Lab Protocol.

**Quantitative evidence for indomethacin’s effect**: Although we have not included a specific graph comparing Purkinje cell survival, we provide robust quantitative evidence through lactate dehydrogenase (LDH) release assays (Figure 2B), which show a 71% reduction in cytotoxicity in serum-free plus indomethacin (SF+I) conditions compared to horse serum (HS) conditions. This reduction in cytotoxicity indicates improved viability across the cerebellar tissue, including Purkinje cells, which are highly sensitive to cytotoxic conditions. Furthermore, the newly added western blot analysis of calbindin expression (Figure 2C) demonstrates higher calbindin levels in SF+I conditions, directly supporting Purkinje cell health and survival. These metrics provide strong quantitative evidence of indomethacin’s beneficial effect on cerebellar tissue, including Purkinje cells.

**Broader impact beyond Purkinje cells**: While the reviewer focuses on Purkinje cells, our protocol’s effectiveness extends to the entire cerebellar tissue, which is critical for studying cerebellar function. The LDH assays indicate reduced cytotoxicity across all cell types, and the high-resolution confocal images in Figure 3 reveal preserved cytoarchitecture across the molecular, Purkinje, and granular layers (e.g., intact Purkinje cell layers in 3D and elaborate dendritic arborization in 3E). This broader preservation supports the protocol’s utility for studying various cerebellar cell populations and their interactions, not limited to Purkinje cells.

**The role of indomethacin in imaging**: Without indomethacin, the cerebellar tissue remains swollen, rendering imaging methods ineffective and hindering detailed morphology. The improved optical clarity observed in SF+I conditions (Figure 2D, brightfield images) enables the acquisition of high-resolution (40x) confocal images in Figure 3, which depict single-cell morphology, including Purkinje cell dendritic arborization. This highlights indomethacin’s vital role in reducing inflammation and swelling, thus facilitating high-quality imaging and morphological analysis.

**Addressing suggested quantification methods**: We appreciate the reviewer’s suggestions to incorporate methods like the cytoarchitecture index (Ayala-Nunez et al., 2019) or dendrite length/surface area (Sherkhane and Kapfhammer, 2017). These methods, which quantify structural features such as cell density or dendritic complexity, are valuable for detailed morphological studies. However, for this protocol paper, our focus is on establishing a reliable method for maintaining cerebellar slice viability, and our current metrics—LDH assays for overall cytotoxicity and calbindin expression for Purkinje cell health—effectively validate this goal. In non-optimized conditions (e.g., HS, SF), high cytotoxicity and tissue swelling, as shown in Figure 2B and 2D, result in extensive cell loss and structural degradation, making detailed morphological quantifications less informative. Our data, particularly the preserved architecture in Figure 3, provide sufficient evidence of indomethacin’s benefits while laying a foundation for future studies to apply advanced quantifications, such as those suggested.

**Improved image quality**: We have addressed the reviewer’s concern regarding image quality by replacing all representative images with higher-resolution versions, aligning with standards observed in recent publications (e.g., MacLeod et al., 2023). Specifically, Figure 3 now includes 40x confocal images that clearly resolve single-cell morphology across the molecular, Purkinje, and granular layers, with panels 3D and 3E highlighting intact Purkinje cell layers and dendritic arborization, respectively. The high-resolution tiled overview in Figure 3A, made possible by the optical clarity under SF+I conditions (Figure 2D), further demonstrates the preservation of cerebellar architecture, including the deep cerebellar nuclei, where present.

Our quantitative data (LDH assays, calbindin western blot) and qualitative data (high-resolution imaging) collectively demonstrate the beneficial effect of indomethacin on cerebellar slice viability, particularly regarding Purkinje cells, while also promoting broader tissue health. The protocol’s capacity to reduce tissue swelling in SF+I conditions facilitates high-quality imaging, thereby addressing the reviewer’s concerns about image standards. We acknowledge the importance of additional quantifications, such as the cytoarchitecture index or dendrite measurements, for future studies and intend to integrate these into subsequent research to characterize cellular health and connectivity further. These revisions, outlined in the Expected Results (pages 5-6) and Materials and Methods (pages 3-5), enhance the manuscript’s evidence and align with its objective of validating a reliable protocol for organotypic cerebellar slice cultures from aged mice.

3. Figure 4 (PI and TCZ experiments):

**Reviewer Comment**: “No conclusions can be drawn in the absence of statistically validated data. The authors should present a graph comparing the number of PI+ cells per unit length in the presence and absence of TCZ.”

We appreciate the reviewer’s comment and agree that further validation of the propidium iodide (PI) and tocilizumab (TCZ) experiments is necessary to draw statistically supported conclusions. However, since these experiments are not essential to our primary goal of validating the organotypic cerebellar slice culture (OCerSC) protocol, we have removed them from the manuscript to maintain focus. The revised manuscript now emphasizes indomethacin’s role in enhancing viability, supported by lactate dehydrogenase (LDH) release assays (Figure 2B), which demonstrate a 71% reduction in cytotoxicity in serum-free plus indomethacin (SF+I) conditions compared to horse serum (HS) conditions, indicating improved overall tissue viability. This is further reinforced by the new western blot analysis of calbindin expression (Figure 2C), showing higher calbindin levels in SF+I conditions, which validates Purkinje cell health as part of the broader cerebellar tissue. Additionally, the enhanced optical clarity in SF+I conditions (Figure 2D) enables high-resolution confocal images in Figure 3, revealing well-preserved morphology across the molecular, Purkinje, and granular layers, which would be obscured by tissue swelling in the absence of indomethacin. This streamlined approach ensures clarity and alignment with the protocol’s core goals of establishing reliable slice viability, as detailed in the Expected Results (pages 5-6).

4. Minor Concerns:
   - Figure 2C:

**Reviewer Comment**: “Figure 2C is useless, as the images are not sufficiently informative… there are no statistically validated data (e.g. measurement of slice area over time). This figure could be deleted.”

We greatly appreciate the reviewer’s feedback and their suggestion to enhance the informativeness of Figure 2C. We have revised this figure, now labeled as Figure 2D, to provide a more effective qualitative guide for readers performing the protocol. The updated bright-field images, enhanced with a thermal lookup table to highlight opacity differences, provide a clear visual representation of tissue health across conditions, particularly with the improved clarity in serum-free plus indomethacin (SF+I) conditions compared to horse serum (HS). **This qualitative readout is designed to help users assess slice viability and optimize culture conditions during the protocol, aligning with the practical goals of a *PLOS ONE* Lab Protocol.** We believe these improvements make Figure 2D a valuable tool for researchers, addressing the reviewer’s concern without necessitating quantitative analysis, which is beyond the scope of this protocol-focused manuscript.

- Figure 2 legend mismatch:

**Reviewer Comment**: “The symbols described in the legend (lines 417 and following) do not correspond to those shown in the figure.”
We have corrected the mismatch between the legend and the symbols in Figure 2, ensuring consistency in the revised manuscript.

- Indomethacin reference and mode of action:

**Reviewer Comment**: “Line 150: a bibliographic reference should be added for indomethacin. The mode of action of this molecule could also be described.”
We have added a reference to indomethacin [31, 35, 36] and expanded the Discussion (pages 6-7) to describe its mechanism as a non-selective cyclooxygenase (COX-1 and COX-2) inhibitor, which reduces prostaglandin synthesis and mitigates inflammation. This addition provides necessary context for its role in our protocol.

- Labeling other neuronal populations:

**Reviewer Comment**: “The proper maintenance of cerebellar cytoarchitecture in culture could also be studied by labeling other neuronal populations in the cerebellar cortex (e.g. anti-parvalbumin to label molecular layer interneurons).”

We sincerely appreciate the reviewer’s insightful suggestion to enhance our understanding of cerebellar cytoarchitecture by labeling additional neuronal populations. In the current protocol, we utilize calbindin immunostaining to highlight Purkinje cells and their axons, complemented by DAPI staining to visualize nuclei, including large neurons in the deep white matter, as detailed in the high-resolution images in Figure 3. This approach provides a comprehensive view of the molecular, Purkinje, and granular layers, as well as the deep cerebellar nuclei when present. While we have not incorporated anti-parvalbumin immunostaining in this study, we recognize its value for labeling molecular layer interneurons (e.g., basket and stellate cells) and plan to explore this in future studies to further characterize the cerebellar cortex. This protocol establishes a robust foundation for such investigations, and the improved imaging quality, enabled by indomethacin’s reduction of tissue swelling (Figure 2D, supports the potential for expanded staining approaches. We thank the reviewer for this suggestion, which enhances the manuscript’s direction for future research.

- LDH assay explanation:

**Reviewer Comment**: “The principle of the LDH assay needs to be explained in greater detail.”

We have expanded the Materials and Methods section (pages 3-5) to provide a detailed description of the LDH assay’s principle, methodology, and application. The assay measures LDH release as an indicator of cell membrane damage, utilizing a bioluminescent reaction quantified at 490 nm. Appropriate citations ([32]) and technical details (e.g., triplicate measurements, storage conditions) have been included to ensure clarity and reproducibility.

# General Comments

- **Protocol on protocols.io**: The step-by-step protocol is available on protocols.io (dx.doi.org/10.17504/protocols.io.q26g71428gwz/v1) and is included as Supporting Information file S1, meeting *PLOS ONE*’s requirements for Lab Protocols.
- **Value of the protocol**: Our protocol addresses a critical gap in the literature by enabling long-term (≥14 days) maintenance of cerebellar slices from aged mice (>6 months), surpassing the typical <7-day viability of adult brain slices. The use of serum-free media with indomethacin reduces cytotoxicity by 71% compared to horse serum conditions (Figure 2B), facilitating studies on age-related cerebellar changes, such as Purkinje cell dysfunction in ataxia and broader tissue health (Discussion, pages 6-7). This improvement is essential, as indomethacin prevents tissue swelling that obscures imaging and morphology in non-optimized conditions.
- **Benchmark data**: We have included benchmark data to validate the protocol’s effectiveness, including:
  - LDH release assays showing reduced cytotoxicity in SF+I conditions (Figure 2B).
  - Western blot analysis quantifying calbindin expression (Figure 2C) has been newly added to confirm the health of Purkinje cells and overall tissue.
- **Revised figures**: The manuscript now includes:
  - Figure 2C (now 2D): Updated bright-field images with a thermal lookup table to qualitatively assess tissue clarity.
  - Figure 3: High-magnification images of cerebellar layers and DCN where present.
- **Integration of calbindin western blot**: The newly added western blot data (Figure 2C) strengthens our evidence of Purkinje cell and overall cerebellar tissue preservation, directly addressing Reviewer #1’s request for neuronal functionality and Reviewer #2’s call for quantitative data. The blot reveals higher calbindin expression in SF+I conditions, reinforcing the protocol’s effectiveness across cell types.
- **Cerebellar nuclei clarification**: We have clarified that the deep cerebellar nuclei (DCN) are present in some parasagittal slices but not in all, depending on slice positioning. Their neurons are not calbindin-positive; however, Purkinje cell axons in the DCN are, as shown in Figure 3. DAPI staining distinguishes DCN neurons, ensuring compliance with the reviewer’s imaging request.

**Conclusion**

We sincerely thank the reviewers and editor for their insightful and constructive feedback, which has significantly improved the manuscript. The revisions—including high-magnification images, quantitative data from LDH and calbindin western blot analyses, and additional references—address all concerns while aligning with *PLOS ONE*’s Lab Protocol standards. We believe the updated manuscript presents a robust and reproducible protocol that adds substantial value to the field of neurobiology, particularly in the study of age-related cerebellar disorders. We hope these changes meet your expectations and look forward to your further guidance.

**Sincerely,**
Jonathan C. Schisler (on behalf of all authors)

*Dear Dr. Schisler,

Thank you for reaching about this issue, and please accept my apologies on behalf of the journal for not getting back to you sooner. 

I'm sorry that there appears to have been a miscommunication on behalf of the reviewers here. Please feel free to respond to these comments in your Response to Reviewer document when you resubmit your manuscript. Please also feel free to cite PLOS One's Lab Protocol Article Template directly in your responses.

I hope this is helpful. If you have any further questions or concerns, please feel free to reach back out.

Kind regards,
Ethan

Ethan Krajewski
Peer Review Operations Specialist*
[*plosone@plos.org*](mailto:plosone@plos.org) *| he/him

Case Number: 08882501*
