# Supplementary material for: A protocol to establish and maintain organotypic cerebellar slice culture (OCerSC) from aged mice
Source: PLoS One. 2026 Apr 17;21(4):e0342373. doi: 10.1371/journal.pone.0342373 (PMC13089748; doi:10.1371/journal.pone.0342373)
Supplement: Attachment — Submitted filename: Almeida et al OCerSC Responses.docx [file pone.0342373.s005.docx]

Dear Editors and Reviewers,

Thank you for the constructive feedback on our manuscript, "A protocol to establish and maintain organotypic cerebellar slice culture (OCerSC) from aged mice" (Manuscript ID: PONE-D-24-43970R1). We appreciate the reviewers' positive comments and have carefully addressed all points raised by the Academic Editor and both reviewers. These revisions improve the manuscript's clarity, rigor, reproducibility, and usefulness as a resource for researchers working with aged cerebellar tissue. Below is a detailed point-by-point response outlining specific changes made, including references to revised figures, tables, and text sections.

# Response to Editor’s Requirements

Changes to the Number and Order of Authors: We thank the Editor for the opportunity to clarify these updates. The authorship changes accurately reflect contributions made during the revision process and fully comply with PLOS ONE and ICMJE authorship criteria.

- **Addition of Michael A. Garris (M.A.G.):** Mr. Garris made significant contributions to new western blot experiments that provided essential data addressing reviewer concerns and improving protocol validation. His involvement took place after the initial review.
- **Addition of Rebekah Sanchez-Hodge (R.S.):** Dr. Sanchez-Hodge provided significant intellectual input by critically reviewing and editing the revised manuscript and helping draft detailed reviewer responses. Her contributions began during revision.
- **Adjustment of Author Order:** The order was revised to better reflect the overall contributions. Michael F. Almeida (M.F.A.) and Kaitlan Smith (K.S.) are now designated co-first authors (marked with *), acknowledging Dr. Almeida's leadership in experimental revisions, data analysis, and response coordination—equal to Ms. Smith's prior contributions. All authors unanimously approved this change.

*All authors have confirmed their approval of these changes through direct submissions to the journal, as required. No disputes exist, and the revised list accurately reflects each individual's contributions.*

# Response to Reviewer #1

1. **Removal of the FS+I (15 days) group in Figure 2B:**

We thank the reviewer for noting this change. The whole 15-day indomethacin treatment group was initially included to compare short- and long-term effects. Results showed no significant additional benefit beyond 5 days, consistent with indomethacin's role in reducing early gliosis and inflammation during slice recovery. To streamline the presentation and emphasize the optimized protocol (indomethacin for only the first 5 days), we have moved this dataset to **Supplementary Figure 1**, with full details in the legend and references in the Results and Methods sections. This change enhances clarity while maintaining transparency.

2. **Differences in LDH data between the original and revised versions:** We appreciate this careful observation. The changes come from:

- **Refined sampling schedule:** Media collections moved to days 1, 2, 4, 8, 12, and 15 (compared to previous days 1, 4, 7, 9, 12, 15), with full replacements at each point. This resets cumulative LDH release, supplies fresh nutrients to support recovery, and results in lower overall levels—indicating improved tissue health in independent replicates.
- **Inherent biological variability in aged tissue:** Organotypic cultures from aged mice exhibit greater variability than those from neonatal mice due to differences in donor age, accumulated oxidative stress, neurodegeneration, and pre-dissection factors (e.g., inflammation). This is well-documented in adult/aged slice models and highlights the importance of using replicates. Ratios (e.g., SF vs. SF+I) stay consistent in both direction and significance, supporting the robustness of the protocol. We included a discussion of these factors in the revised Discussion section for context.

We believe these explanations fully address concerns about reproducibility.

3. **Authorship changes:** As detailed in the Editor’s response above, all modifications were consensual and reflect revision contributions. Confirmations have been submitted to the journal.
4. **PI staining and potential LDH misinterpretation:** We agree that propidium iodide (PI) is a gold standard for visualizing cell death. This protocol paper focuses on establishing long-term, viable OCerSC from aged mice. Detailed mechanistic cell death studies using PI (including prior TCZ validation) are reserved for future applications of the method and are thus not included here. We note PI as recommended for extensions in the revised Discussion.

For LDH reliability: We implemented rigorous controls (media-only blanks subtracted; serum-free/serum baseline measurements; Triton X-100 normalization to % maximal release; antibiotics to prevent contamination). These measures reduce false positives and negatives and ensure precise cytotoxicity measurement. LDH is commonly used in organotypic slices to track cumulative release, complementing direct methods such as PI. We believe this approach is sufficient for protocol validation.

**Minor points:**

- **AUC for LDH:** Added as Supplementary Table 1, with 2-way ANOVA results referenced in the text and Figure 2 legend.
- **Heat maps (Figure 2D):** Legend expanded with explicit time points and interpretive notes on viability gradients (see revised Figure 2 legend).
- **Individual values in Figure 2B:** Scatter plots with all biological replicates overlaid on the fit curve (see revised Figure 2B).
- **SF abbreviation:** Changed to "Stain-Free total protein" throughout to eliminate confusion.
- **Cerebellar nuclei:** Clearly labeled in IF images (see revised Figure 3A and legend).

*We believe these changes fully address the reviewer's concerns*

# Response to Reviewer #2

**Minor concerns:**

- **Missing immunostaining under detrimental conditions (HS and/or HS+I):** We agree that comparing calbindin staining across all conditions would be helpful. However, the HS and HS+I conditions lead to significant tissue degeneration and poor flattening by around 7 DIV, resulting in high background and unreadable immunofluorescence. This aligns with previous research on adult cerebellar slices and underscores the need for serum-free, early-indomethacin conditions to preserve cytoarchitecture. We addressed this limitation in the revised Discussion section.
- **Reason for using Hibernate-A medium:** Hibernate-A (supplemented) was chosen for dissection and slicing because it supports short-term maintenance of adult neural tissue at ambient CO₂ without carbogen gassing, thereby reducing manipulation stress, excitotoxicity, and osmotic shifts that are critical for aged cerebellar viability. It provides stable physiological buffering and nutrients during benchtop handling, unlike traditional carbogen-dependent media. This aligns with optimized adult protocols, such as those for the hippocampus, and contributes to high survival rates. We expanded the rationale in the revised Methods section.

*We believe these revisions fully address the comments and improve the manuscript. Thank you again for your time and expertise—we look forward to your final decision.*
